# Serum levels of miR-21, miR-23a, miR-142-5p, and miR-126 in chronic failure with reduced ejection fraction: a case-control study

**DOI:** 10.3389/fcvm.2025.1529451

**Published:** 2025-05-15

**Authors:** Lukas Evin, Radka Sigutova, Patrik Sulc, Eliska Kufova, Marian Branny, Jan Vaclavik, David Stejskal

**Affiliations:** ^1^ Department of Internal Medicine and Cardiology, University Hospital Ostrava, Ostrava, Czechia; ^2^ Research Center for Internal and Cardiovascular Diseases, Faculty of Medicine, University of Ostrava, Ostrava, Czechia; ^3^ Institute of Laboratory Medicine, University Hospital Ostrava, Ostrava, Czechia; ^4^ Institute of Laboratory Medicine, University of Ostrava, Ostrava, Czechia

**Keywords:** microRNA, heart failure, HFrEF, miR-21p, miR-23a-3p, miR-142-5p, miR-126-3p

## Abstract

**Background:**

MicroRNAs (miRNAs) are small non-coding RNA molecules that function as gene regulators in physiological processes, including proliferation, differentiation, and apoptosis. Various microRNAs have been linked to pathophysiological events associated with heart disease. In this case-control study, we investigated the levels of human miR-21, miR-23a, miR-142-5p, and miR-126 among heart failure patients with reduced ejection fraction (HFrEF), compared to healthy control participants.

**Methods:**

We prospectively enrolled clinically stable patients with heart failure (HF) and left ventricle ejection fraction (LVEF) ≤ 40%, and healthy individuals. MicroRNAs were analyzed from venous blood, using a microRNA enzymatic immunoassay (miREIA) method. Plasma miRNA levels were compared between HFrEF patients and healthy individuals, using non-parametric tests.

**Results:**

We enrolled 73 patients with HFrEF (86% males, mean age: 66.3 ± 10.7 years) and 99 healthy subjects (36% males, mean age: 44.7 ± 15.9 years). All four assayed miRNAs exhibited significantly higher median levels in heart failure patients compared to healthy controls: miR-21p, 243 pmol·l^−1^ vs. 14 pmol·l^−1^; miR-23a-3p, 705 pmol·l^−1^ vs. 119 pmol·l^−1^; miR-142-5p, 1,695 pmol·l^−1^ vs. 146 pmol·l^−1^; and miR-126-3p, 528 pmol·l^−1^ vs.21 pmol·l^−1^ (*P* ≤ 0.001 for all). The analyzed miRNA levels did not differ according to age, weight, height, or body mass index. No miRNA levels correlated with NTproBNP levels.

**Conclusion:**

Our findings revealed that the levels of miR-21-5p, miR-23a-3p, miR-142-5p,and miR-126-3p were significantly higher among HFrEF patients compared to healthy controls. Further exploration of these miRNAs may lead to new diagnostic, prognostic, and therapeutic options for HF patients.

## 1 Introduction

MicroRNAs (miRNAs) are small non-coding RNA molecules that do not serve as templates for protein synthesis, but rather function as gene regulators in physiological processes, such as proliferation, differentiation, and apoptosis (1). MiRNAs are detected extracellularly in blood samples. They can be packaged into exosomes, microvesicles, and apoptotic vesicles and transported in the blood. Extracellular miRNAs are highly stable and resistant to degradation by circulating ribonucleases (2).

Various microRNAs have been linked to pathophysiological events associated with heart disease (HD). Notably, miR-21-5p plays important roles in the proliferation and apoptosis of vascular smooth muscle cells, the growth and death of cardiac cells, and the function of cardiac fibroblasts (3). Several small studies have reported increased circulating miR-21 levels in HF patients compared with controls (4, 5). Another study demonstrated that circulating miRNA-21 levels are correlated with HF diagnosis and severity, and the prognosis and re-hospitalization rate among patients with HF (6). Another miRNA, miR-23a, promotes cardiomyocyte apoptosis by inhibiting mitochondrial superoxide dismutase (MnSOD) expression (7). Myocardial biopsy specimens from patients with recent heart failure have exhibited significant up-regulation of miRNA-21 and miRNA-23, compared to myocardial tissue from patients without HF (8).

Additionally, miR-126-3p has been reported to be an independent prognostic factor for patients with HF (9). Another study demonstrated that circulating miR-21 and miR-126 levels increase with HF decompensation and were associated with better survival and rehospitalization rates (10). In a Japanese study, miR-126 plasma levels were negatively correlated with age and NYHA class among patients with HF (11). Jiao et al. reported that miR-126-3p and miR-142-5p are potential biomarkers for the diagnosis of dilated cardiomyopathy (DCM) in pediatric patients (12). In their next study, these investigators compared the circulating miRNA levels in patients with isolated diastolic dysfunction, compensated dilated cardiomyopathy, and decompensated congestive HF. They reported MiR-142-3p downregulation in the DCM and DCM-HF groups but not in patients with isolated diastolic dysfunction (13).

Overall, the available data indicate a pathogenic role of the described miRNAs in HF development, and that increased levels of these miRNAs are observed in failing hearts in humans and animal models. Therefore, in the present study, we explored associations between HFrEF and the levels of four circulating miRNAs (miR-21-5p, miR-23a-3p, miR-142-5p, and miR-126-3p).

## 2 Methods

For this study, we enrolled patients who met the following inclusion criteria: chronic heart failure with left ventricular ejection fraction (LVEF) ≤ 40%, and age between 18 and 80 years. HF was diagnosed according to the guidelines of the 2012 European Society of Cardiology (14). The exclusion criteria for the patient group were recent (within 3 months) myocardial infarction or percutaneous coronary intervention, recent decompensation of chronic heart failure, chronic kidney disease requiring hemodialysis, known malignancy, serum creatinine level >450 µmol·l^−1^, or signs of acute or chronic inflammation with C reactive protein (CRP) > 20 mg·l^−1^.

**Figure 1:**
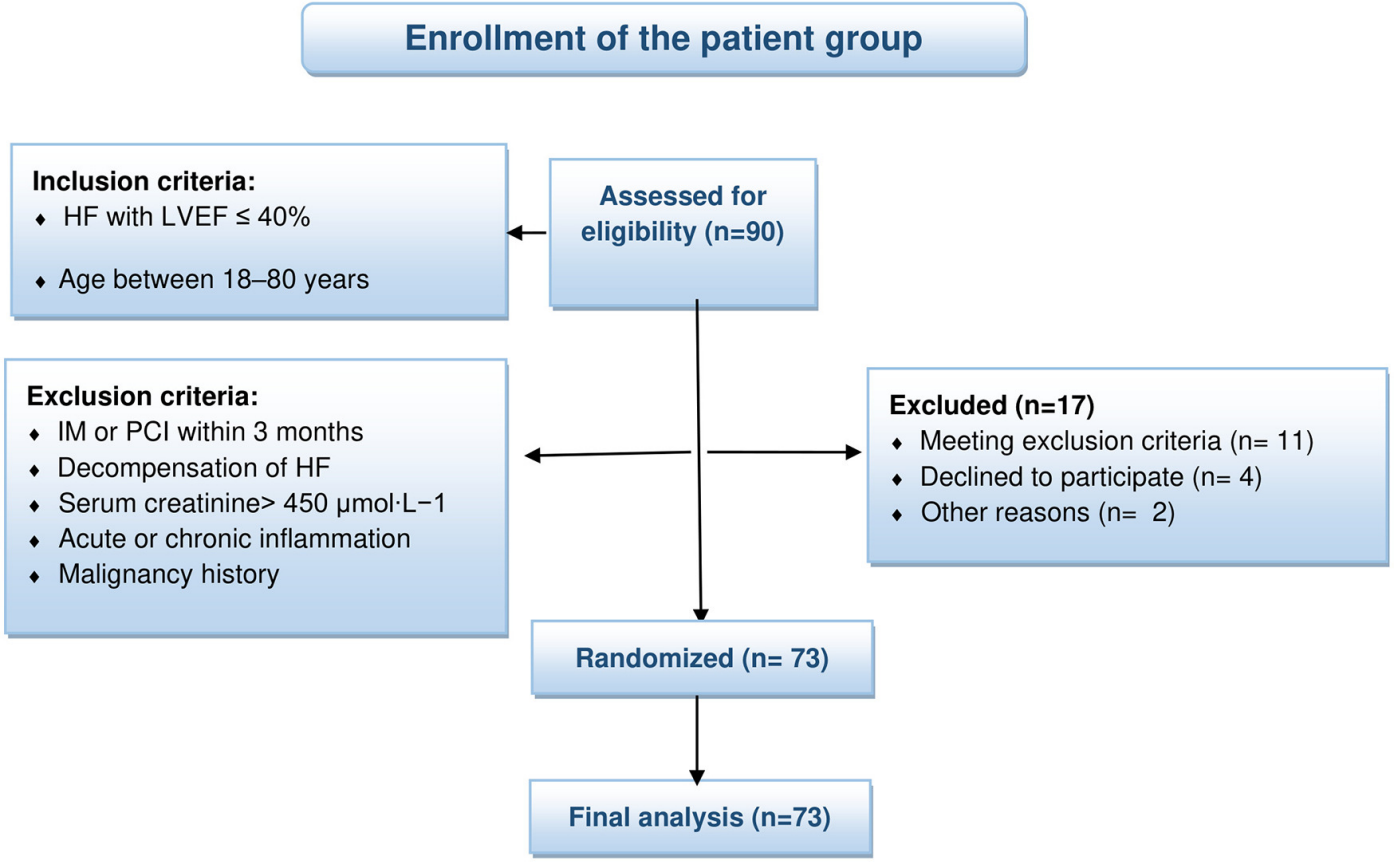
Flow diagram for patient group.

Among individuals who were hospitalized between January 2019 and January 2022, a total of 90 patients met the inclusion criteria for this study, signed an informed consent form, and were subsequently recruited into the study. All patients underwent echocardiography (ECHOKG) at our department. Among the 90 recruited patients, 17 met exclusion criteria or decided to quit the study, and were removed from the patient group and study (see Figure 1).

**Figure 2:**
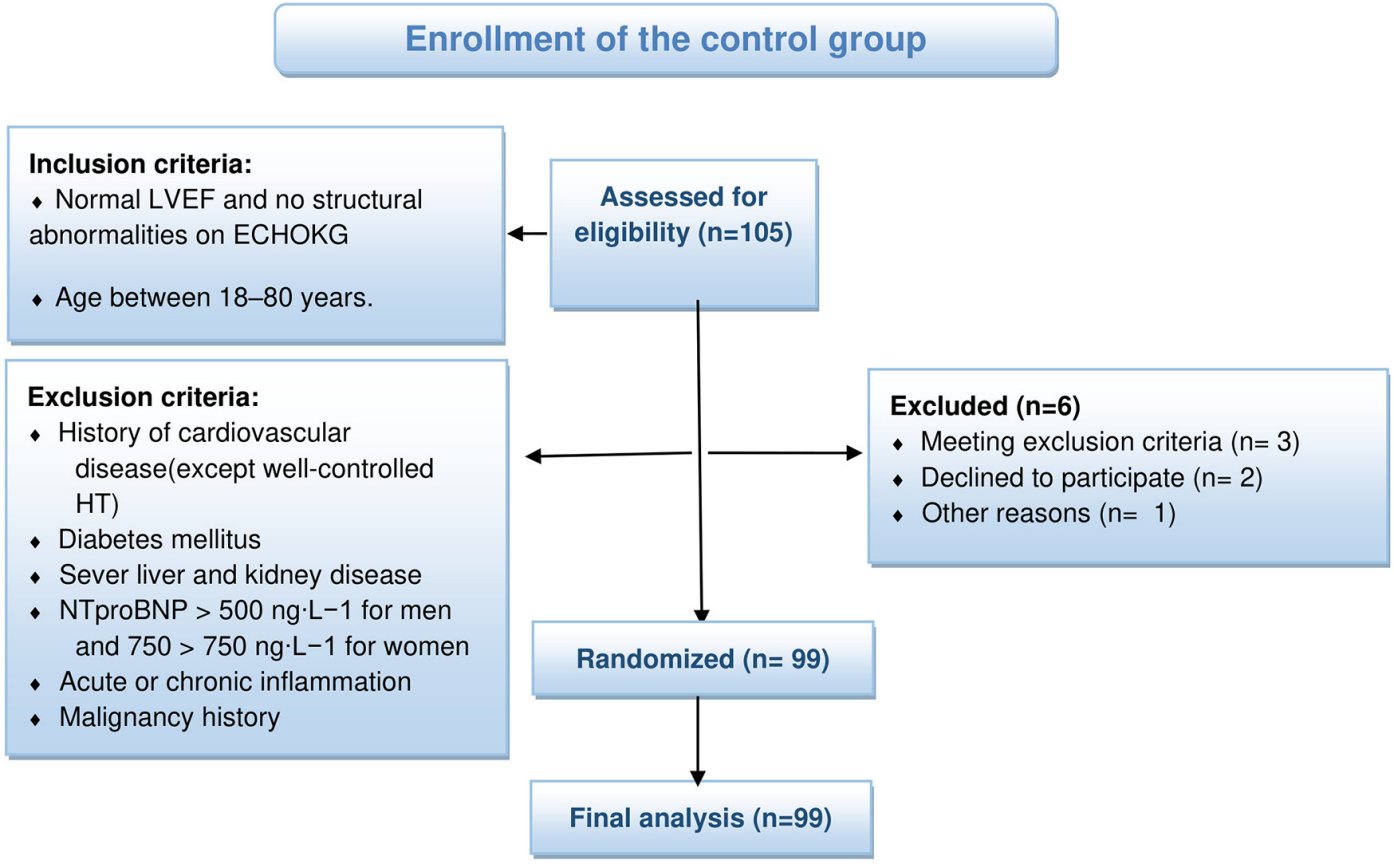
Flow diagram control group.

For the control group, we recruited healthy individuals, with normal LVEF and no structural cardiac abnormalities, and age between 18 and 80 years. Exclusion criteria were as follows: history of any cardiovascular disease (except well-controlled arterial hypertension), diabetes mellitus, severe liver and kidney disease (checked by initial laboratory testing), NTproBNP >500 ng·l^−1^ for men and 750 ng·l^−1^ for women, known malignancy, or signs o acute or chronic inflammation with CRP >20 mg·l^−1^. Among 105 recruited healthy individuals who met the inclusion criteria, 6 met exclusion criteria or decided to quit the study, and were removed from the control group and study (see Figure 2).

This study was approved by the Research and Ethics Committee of the University Hospital of Ostrava, and was conducted in accordance with the Helsinki Declaration. All participants gave their signed informed consent.

### 2.1 Biochemical analysis

Whole blood samples were obtained from all study participants. Samples were collected in S-Monovette serum-gel tubes (4.9 ml; Sarstedt AG & Co. KG, Germany) to evaluate classic biochemical markers (CRP, GLU, NTproBNP, etc.). These biomarkers were measured from the serum immediately after centrifugation, at the certified laboratory of the Department of Clinical Biochemistry, Ostrava University Hospital. We also obtained a whole blood sample from each participant into PAXgene® Blood RNA tubes (Vacutainer® PAXgene, PreAnalytiX® GmbH, A Qiagen/BD Company, Switzerland). These samples were initially stored upright at room temperature for 2 h, and then frozen at −20°C for 24 h, and finally stored at −80°C until miRNA isolation.

Target miRNA isolation was performed using an RNA isolation kit (batch No.RIK001; BioVendor Laboratory Medicine Corp.) at the Institute of Laboratory Medicine, Department of Clinical Biochemistry, University Hospital of Ostrava. The target miRNA concentration, as measured using a microRNA enzymatic immunoassay (miREIA), can be affected by the efficiency of RNA isolation. Therefore, we also monitored isolation efficiency. The exogenous control used for normalization was cel-miR-39-3p from the RNA isolation kit (batch No. RDM0000C; BioVendor Laboratory Medicine Corp.), at a concentration of 10 nmol·l^−1^. RNA purification was performed according to the manufacturer's protocol.

Selected specific miRNAs were assayed using the MiREIA immunochemical method (BioVendor Laboratory Medicine Corp., Brno, Czech Republic). This method uses a biotinylated DNA probe complementary to the target miRNA. After hybridization, the resulting DNA/miRNA heterohybrids in the wells of the plate are recognized by a specific monoclonal antibody immobilized on the surface of the wells. After the addition of streptavidin conjugated to the enzyme HRP (Horse Radish Peroxidase) and subsequent visualization of the resulting complexes, a chromogenic substrate (TMB, tetramethylbenzidine) is added. After the addition of a stopping solution, the absorbance at 450 nm is measured, which corresponds to the concentration of the target miRNA.

The determined concentration of each miRNA was subjected to technical data normalization. To this end, we calculated an efficiency ratio of isolation for each sample, based on the division of an expected concentration of the exogenous control (cel-miR-39-3p) by the concentration determined by measuring the miRNA target. The concentration of each target miRNA was then multiplied by this ratio.

### 2.2 Statistical analysis

Numerical variables are expressed as median and interquartile range (IQR). Categorical variables are presented as frequency and relative frequency, as percentages. Defined groups were compared using the t-test for independent samples (for normal distributions) or the Mann–Whitney test (for non-normal distributions). The Chi-square test of independence was applied for contingency tables. To evaluate the relationships between two numerical variables, we used Pearson's or Spearman's rank correlation coefficients and their test of significance. All statistical analyses were performed using IBM SPSS, version 29. The significance level was set to 0.05.

## 3 Results

We evaluated data from 99 control participants (36% male, mean age: 44.7 ± 15.9 years) and 73 patients (86% male, mean age: 66.3 ± 10.7 years). The two groups significantly differed in both the sex distribution and mean age. Details are presented in Table 1. We performed correlation analyses within the patient and control groups, which revealed no or very low significant correlations between the miRNAs and participant age, weight, height, and BMI, and no significant differences within groups (Table 2). Comparison of basic biochemical findings between groups corresponded with the nature of the two groups (healthy vs. sick individuals). Further details are shown in Table 3. We examined the correlation between the studied miRNAs and NTproBNP levels, and found no or very low correlations, and no significant differences within groups (Table 4). In patient group there were 56 patients with ischemic cardiomyopathy. We compared ischemic and non-ischemic patient for miRNA analysis and there was no significant difference. (miR-21-5p, *p* = 0.215; miR23a-3p, *p* = 0.715; miR-142-5p, *p* = 0.604; miR-126-3p, *p* = 0.481).

**Table 1:** Basic demographic and anthropometric data.

Parameters	Controls	Patients	*P* value
Age, years	44.7 ± 15.9	66.3 ± 10.7	≤0.001
Sex	36% male	86% male	≤0.001
Weight, kg	77 ± 18.9	88.6 ± 19.2	0.003
BMI, kg/m^2^	26. ± 7	28 ± 5	0.011
Height, cm	171 ± 15.4	175 ± 7.6	0.014

**Figure 3:**
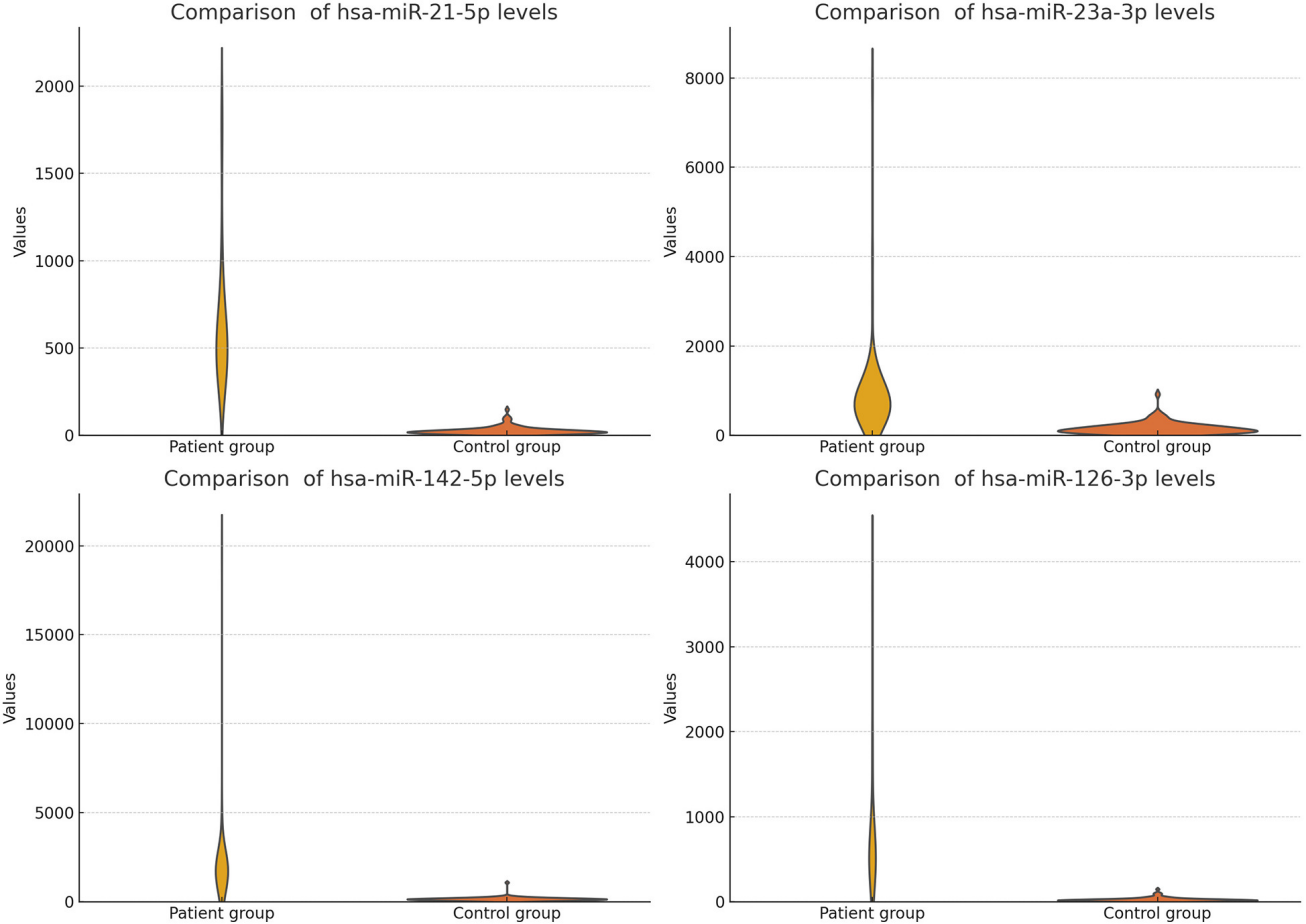
Comparison of analyzed microRNA levels in the patient group with HF, and the healthy control group (unfiltered).

**Table 2:** Correlations of basic demographic and anthropometric data.

Parameters		Spearman's correlations							
		hsa-miR-21-5p		hsa-miR-23a-3p		hsa-miR-142-5p		hsa-miR-126-3p	
		Control	Patient	Control	Patient	Control	Patient	Control	Patient
Age	Correlation Coef.	−0.24	−0.01	−0.19	0.038	−0.08	0.159	−0.12	−0.002
	*P* value	0.015	0.922	0.059	0.75	0.413	0.195	0.251	0.987
Weight, kg	Correlation Coef.	0.007	0.029	−0.005	0.08	0.041	0.079	−0.081	0.111
	*P* value	0.949	0.807	0.964	0.505	0.684	0.523	0.424	0.354
Height, cm	Correlation Coef.	0.184	0.113	0.041	0.028	0.102	−0.046	0.132	0.028
	*P* value	0.068	0.341	0.690	0.812	0.313	0.707	0.193	0.813
BMI, kg/m^2^	Correlation Coef.	−0.047	0.009	−0.009	0.105	0.022	0.086	−0.146	0.073
	*P* value	0.942	0.942	0.929	0.38	0.832	0.487	0.15	0.542

**Table 3:** Overview and comparison of mean values of biochemical parameters.

Biochemical parameters	Controls	Patients	*P* value
Leukocytes, 10^9^·l^−1^	6.0 ± 2	8.0 ± 2.5	0.001
Hemoglobin, g·l^−1^	139.2 ± 12	136.7 ± 15	0.236
Hematocrit	0.4 ± 0.03	0.4 ± 0.0	0.633
Thrombocytes, 10^9^·l^−1^	249.5 ± 57	209.1 ± 60	0.001
Urea, mmol·l^−1^	4.8 ± 1.2	6.2 ± 2.7	0.001
Creatinine, mmol·l^−1^	74.4 ± 16	96.1 ± 27	0.001
ALT, ukat·l^−1^	0.4 ± 0.29	0.5 ± 0.3	0.337
AST, ukat·l^−1^	0.4 ± 0.15	0.4 ± 0.1	0.533
Glucose, mmol·l^−1^	5.2 ± 0.72	6.8 ± 2.2	0.001
CRP, mg·l^−1^	2.2 ± 2.5	6.9 ± 6.7	0.001
NTproBNP, ng·l^−1^	74.4 ± 73	2,790 ± 4,468	0.001
HbA1c, mmol·mol^−1^	35.2 ± 4.3	48.3 ± 13.2	0.001

**Table 4:** Correlations of NTproBNP and measured MiRNA values.

Parameter		Spearman's Correlations							
		hsa-miR-21-5p		hsa-miR-23a-3p		hsa-miR-142-5p		hsa-miR-126-3p	
		Control	Patient	Control	Patient	Control	Patient	Control	Patient
NTproBNP [ng.l^−1^]	Correlation Coef.	−0.138	0.023	−0.065	0.205	−0.047	0.128	0.004	−0.04
	*P* value	0.174	0.848	0.527	0.084	0.641	0.297	0.972	0.74

**Figure 4:**
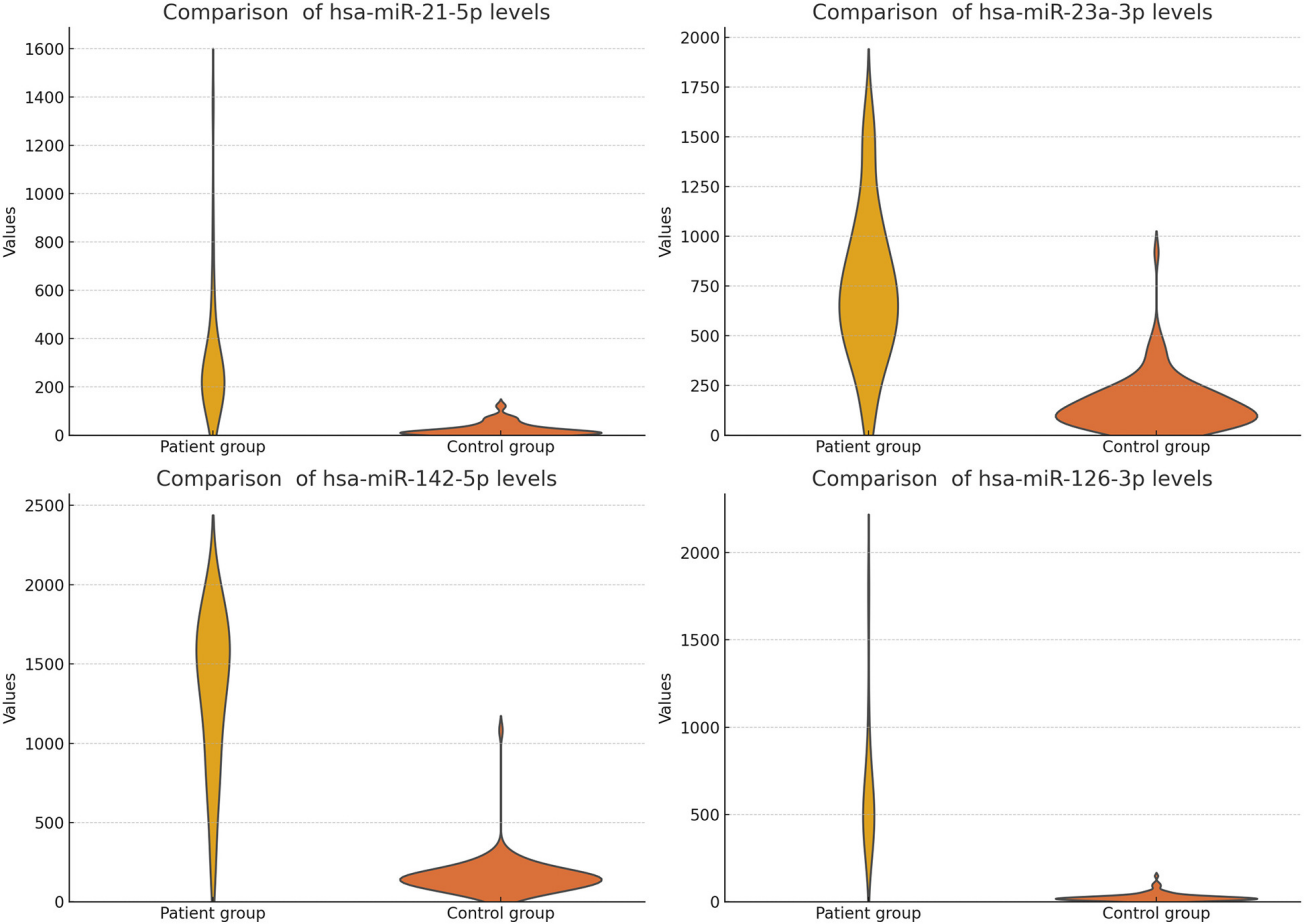
Comparison of analyzed microRNA levels in the patient group with HF, and the healthy control group (filtered).

The study findings are summarized in Table 5 and Figures 3, 4 (We removed values over 2,000 from patient group for better visuality). All analyzed miRNAs (miR-21-5p, miR-23a-3p, miR-142-5p, and miR-126-3p) exhibited significantly higher levels in patients with chronic stable HF, compared to in the healthy control group (*P* < 0.001).

**Table 5:** Measured MiRNA values of patient and control group.

Parameter	*N*		Median, pmol·l^−1^		IQR		*P* value
	Control	Patient	Control	Patient	Control	Patient	
hsa-miR-21-5p	99	73	14	243	6–33	181–327	≤0.001
hsa-miR-23a-3p	96	73	119	705	70.5–203	507–992	≤0.001
hsa-miR-142-5p	99	69	146	1,695	104–187	1,278–2,288	≤0.001
hsa-miR-126-3p	99	73	21	528	13–31	424–776	≤0.001

## 4 Discussion

Based on increasing recognition that miRNAs play roles in cardiovascular diseases, here we aimed to investigate the potential use of circulating miRNAs as biomarkers in HF. We performed a comprehensive miRNA study using whole blood samples from patients of European ethnicity. Compared to prior studies, our investigation included the largest number of samples (*n* = 171 for each miRNA) and largest number of analyzed miRNAs (miR-21-5p, miR-23a-3p, miR-142-5p, and miR-126-3p) from patients with HF with reduced ejection fraction. Other large miRNA studies have focused on HF in general, or have included patients with HF and ejection fraction <45% (10, 15, 16). Our present results revealed that circulating levels of miR-21-5p, miR-23a-3p, miR-142-5p, and miR-126-3p were significantly higher in HF patients with reduced EF compared to healthy controls.

Previous research in a mouse model showed that *in vivo* blockade of miR-21, using a specific antagomir, resulted in decreased cardiac ERK-MAP kinase activity, thereby inhibiting interstitial fibrosis and suppressing cardiac dysfunction (17). These findings suggest that miRNAs may also be targeted for treatment of heart disease. Our present findings confirmed that miR-21 levels were higher in patients with HFrEF compared to healthy controls.

Our data also confirmed the upregulation of circulating miRNA-21-5p in HFrEF. This upregulation has previously been described in heart tissues (8) and plasma (4, 5). Moreover, this phenomenon has been described in DCM, but not in hypertrophic cardiomyopathy. The available data suggest that miRNA-21-5p could be a promising marker for predicting patient prognosis and risk of rehospitalization (10). We plan to continue follow-up in our patient group, to confirm these data on a larger scale.

To date, little data have been available about miR-126 and heart failure. Here we confirmed increased levels of this microRNA in HF patients compared to healthy controls. It is possible that the levels of circulating miR-21 and miR-126 could be a protective factor for HF patients. One prior study reported that increased miR-21 and miR-126 levels at the time of clinical compensation were associated with better 24-month survival, and a longer period without rehospitalization, compared to patients with low levels of these miRNAs (10).

Increased levels of MiRNA-23a-3p have previously been observed in pressure-induced cardiac hypertrophy (18) and cardiac cell apoptosis (7). Both of these mechanisms play a role in heart failure. We observed increased miRNA-23 levels in our HFrEF group, which could play an important role in the pathogenesis of heart failure with reduced ejection fraction.

We found only a few previous clinical reports regarding the role of miR-142-5p in heart failure among adult patients. The available studies include one investigation among pediatric patients (12), a study of dilated cardiomyopathy and diastolic dysfunction (13), an analysis of inflammatory cardiomyopathy (19), and a small study of 15 patients (20). In our present study, we found increased levels of miR-142-5p in patients with HFrEF, which suggests new avenues for further research regarding this miRNA in HF patients.

Our final examined marker was miR-126-3p, which has been previously examined in pediatric patients with HF (12), and in HF and atrial fibrillation (AF) (21). Prior studies have reported increased levels of this miRNA in patients with low ejection fraction. Qiang et al. suggested that miR-126-3p could be a promising independent prognosis factor among patients with HF (9).

In our present study, we used full blood samples for miRNA analysis. Blood samples are most frequently used for diagnostics in routine clinical practice, because they can be easily stored, transported, frozen, and further analyzed. Here we used whole blood for miRNA dentification because it represents the most complete pool of blood fractions. This enables detection of the complete library of miRNAs, along with other parameters and markers, in one blood sample, which can be used for the initial screening. Furthermore, higher levels of miRNAs have been obtained from whole blood compared with serum or plasma (22).

Circulating miRNAs are released into blood both actively and passively (after cell death), and thus may serve as biomarkers of myocardial injury. To minimize the effect of myocardial injury, here we enrolled patients with stable compensated HF, who had not experienced any heart failure decompensation, myocardial infarction, or PCI in the previous 3 months. The average NYHA class in our patients was 2.2, and the mean NTproBNP level was 2,875 ng·l^−1^.

Notably, the NTproBNP levels in our HF patients and controls were not correlated with the levels of the studied miRNAs. Thus, these microRNAs could provide additional information about these patients. Some studies have shown that miRNA levels can be useful for risk stratification and prognosis (9, 10).

Our control group was well-selected, with no or few comorbidities. Only a few of our healthy control participants (17%) had arterial hypertension, which was treated by a maximum of one or two antihypertensive drugs. This setting was ideal for the initial analysis and profiling of miRNAs. The next step would be to include a more morbid control group with diabetes mellitus, obesity, and AF, and treated with more drugs, to obtain a larger picture of the factors associated with these miRNA levels. Since miRNAs may be involved in several pathways, they could be influenced by other diseases, which remains to be examined. Malignancies are one of the greatest focuses in the field of miRNA research. Many miRNAs have been found to play important roles in cell proliferation, differentiation, and apoptosis (1). Notably, these processes are also important in the development of HF, which suggests possible common pathways for the presently investigated miRNAs. Each of the examined markers (miR-21-5p, miR-23a-3p, miR-142-5p, and miR-126-3p) has previously been studied and demonstrated to play a role in malignancies (23–26). Prior studies have demonstrated high serum levels of these miRNAs in patients with malignant tumors; therefore, our current study excluded any patients with known malignancy, such that malignancies should not affect our present results.

MicroRNAs appear to play an important role in cardiology and heart failure. Accumulating evidence, including that from the present study, supports their correlations with the pathophysiology and development of heart failure. These new markers are not just promising as diagnostic tools, but could also provide prognostic information and be targets of future heart failure treatment. There remains a need for more systematic research of the selected miRNAs, to confirm these findings and implement their use in clinical settings.

### 4.1 Limitations

Our sample was unbalanced for sex—exhibiting male predominance in the heart failure group, and female predominance in the healthy volunteer group.

## 5 Conclusion

Our study demonstrated statistically higher levels of miR-21-5p, miR-23a-3p, miR-142-5p, and miR-126-3p in HF patients with reduced EF compared to in healthy controls. Further exploration of these miRNAs may lead to new diagnostic, prognostic, and therapeutic possibilities in HFrEF patients.

## Data Availability

The datasets presented in this study can be found in online repositories. The names of the repository/repositories and accession number(s) can be found in the article/Supplementary Material.
